# *Viroporins*: Structures and Functions beyond Cell Membrane Permeabilization

**DOI:** 10.3390/v7102866

**Published:** 2015-09-29

**Authors:** José Luis Nieva, Luis Carrasco

**Affiliations:** 1 Biophysics Unit (CSIC, UPV/EHU) and Biochemistry and Molecular Biology Department, University of the Basque Country (UPV/EHU), P.O. Box 644, 48080 Bilbao, Spain; 2 Centro de Biología Molecular Severo Ochoa (CSIC, UAM), c/Nicolás Cabrera, 1, Universidad Autónoma de Madrid, Cantoblanco, 28049 Madrid, Spain

Viroporins represent an interesting group of viral proteins that exhibit two sets of functions. First, they participate in several viral processes that are necessary for efficient production of virus progeny. Besides, viroporins interfere with a number of cellular functions, thus contributing to viral cytopathogenicity. Twenty years have elapsed from the first review on viroporins [1]; since then several reviews have covered the advances on viroporin structure and functioning [2,3,4,5,6,7,8]. This Special Issue updates and revises new emerging roles of viroporins, highlighting their potential use as antiviral targets and in vaccine development.

*Viroporin structure*. Viroporins are usually short proteins with at least one hydrophobic amphipathic helix. Homo-oligomerization is achieved by helix–helix interactions in membranes rendering higher order structures, forming aqueous pores. Progress in viroporin structures during the last 2–3 years has in some instances provided a detailed knowledge of their functional architecture, including the fine definition of binding sites for effective inhibitors. A prominent example is that of the Hepatitis C virus p7 viroporin (see structures recently deposited in the Protein Data Bank (PDB) with entries: 3ZD0; 2M6X; 2MTS). New high-resolution structures have also advanced our understanding of the influenza virus M2 viroporin function and inhibition (see for instance structure with PDB entry 2MUV). In addition, new structural models for viroporins derived from other important human pathogens, such as Severe Acute Respiratory Syndrome (SARS) Coronavirus E and the Respiratory Syncytial Virus (RSV) small hydrophobic (SH) proteins, have been made available in the literature [9,10,11].

Based on the number of transmembrane domains (TMD) and the topology adopted in membranes, viroporins have been classified in two groups [4]. This categorization may also reflect the existence of different additional functions in each class, which can be modulated by the physicochemical landscape of the bilayer and the cell physiological factors evolving along the infectious cycle. Thus, viroporins may adapt to specific membrane environments altering the permeability of different compartments, and alter cell physiology by interacting with distinct sets of proteins (see below). Capacity for insertion into membranes according to different mechanisms may also condition these effects. Class I viroporins, composed of a single TMD, are assumed to insert co-translationally into the endoplasmic reticulum (ER) membrane using the translocon machinery. However, Class II viroporins containing two TMDs may insert following more complex mechanisms, including the insertion of a helical hairpin that is stabilized through inter-helical electrostatic interactions, or a post-translational insertion, with or without assistance of the translocon [12,13].

Finally, additional groups should be created for more complex viroporins, as is the case for rotavirus non-structural protein (NSP)-4, which contains three hydrophobic segments and should belong to Class III [14].

*Viroporin functioning*. Viroporins are multifaceted proteins that exert pleiotropic effects on several viral and cellular processes. The activity of viroporins is not only due to the formation of transmembrane pores, but also to their capacity to interact with other viral and cellular proteins. This protein–protein interaction varies according to the particular viroporin analysed; for instance Viral Protein Unique (Vpu) interacts with CD4 receptor and tetherin (BST2), thereby facilitating the budding of virus particles. The main activity of viroporins is to promote virus assembly and exit from infected cells. In general, viruses lacking the viroporin gene are very much weakened and the production of progeny viruses is very low. However, these viruses are still competent to enter cells.

A number of new emerging roles of viroporins are the object of intensive research. Thus, viroporins induce ER stress leading to increased concentrations of cytoplasmic calcium and also alter cell autophagy [15,16]. Disease progression is stimulated by viroporins due to activation of NOD-like receptor family, pyrin domain-containing 3 (NLRP3) inflammasome, thereby modulating the innate immune response [17]. Participation of viroporins in oncogenic processes has been documented by the action of E5 from human papillomavirus, the first oncogenic viroporin described [18].

*New viroporins*. Although viroporins have been mainly identified in RNA viruses, more recently these proteins have also been found in small DNA viruses. Most probably, the number of viroporins will increase in the foreseeable future. In this issue, particular attention is given to coronavirus E protein and to the small hydrophobic (SH) protein from paramixoviruses. Discovery of new viroporins is initially achieved by analysis of their primary sequence and by their ability to increase membrane permeability, as was the case for the aforementioned proteins [19,20,21,22,23].

*Antiviral compounds and vaccine development*. An important aspect of viroporins is their contribution to develop antiviral agents. Thus, high throughput screenings using p7 from hepatitis C virus have identified a number of specific inhibitors [24]. These inhibitors could be of interest, in combination with other antiviral compounds, in order to prevent the appearance of resistant viruses. In addition, these compounds constitute useful tools to study viroporin function. Finally, animal viruses lacking the viroporin gene represent an interesting source for the development of vaccines. Since the step being blocked in the life cycle is the egress of new virus particles, these viruses will be competent to enter into cells and express the rest of their genes [25].
